# A *Caenorhabditis elegans* Zinc Finger Transcription Factor, *ztf-6*, Required for the Specification of a Dopamine Neuron-Producing Lineage

**DOI:** 10.1534/g3.117.300132

**Published:** 2018-01-04

**Authors:** Maria Doitsidou, Gregory Minevich, Jason R. Kroll, Gwen Soete, Sriharsh Gowtham, Hendrik C. Korswagen, Jeroen Sebastiaan van Zon, Oliver Hobert

**Affiliations:** * Department of Biochemistry and Molecular Biophysics, Columbia University Medical Center New York, NY 10032; ** Department of Biological Sciences, Howard Hughes Medical Institute, Columbia University, New York, NY 10027; † Centre for Discovery Brain Sciences, University of Edinburgh, EH8 9XD, UK; ‡ AMOLF, Amsterdam, 1098 XG, The Netherlands, 3584 CT Utrecht, The Netherlands; § Hubrecht Institute, 3584 CT Utrecht, The Netherlands

**Keywords:** ztf-6, Zing finger transcription factor dopaminergic neurons, PDE, postdeirid lineage C. elegans, mediator complex lineage analysis, mutant screen report

## Abstract

Invertebrate and vertebrate nervous systems generate different types of dopaminergic neurons in distinct parts of the brain. We have taken a genetic approach to understand how the four functionally related, but lineally unrelated, classes of dopaminergic neurons of the nematode *Caenorhabditis elegans*, located in distinct parts of its nervous system, are specified. We have identified several genes involved in the generation of a specific dopaminergic neuron type that is generated from the so-called postdeirid lineage, called PDE. Apart from classic proneural genes and components of the mediator complex, we identified a novel, previously uncharacterized zinc finger transcription factor, *ztf-6*. Loss of *ztf-6* has distinct effects in different dopamine neuron-producing neuronal lineages. In the postdeirid lineage, *ztf-6* is required for proper cell division patterns and the proper distribution of a critical cell fate determinant, the POP-1/TCF-like transcription factor.

The relevance of dopamine neurons for human neurological disease has spurred intensive efforts to identify regulators of dopamine neuron differentiation across the animal kingdom (Abeliovich and Hammond 2007; Smidt and Burbach 2007) . One intriguing issue of dopaminergic fate specification is that dopaminergic neurons are generated in distinct parts of the vertebrate nervous system, suggesting that distinct patterning mechanisms may be employed to specify dopaminergic neurons. As in vertebrates, the nervous system of the hermaphroditic *Caenorhabditis elegans* also contains a lineally diverse set of dopaminergic neurons. Specifically, there are eight dopaminergic neurons that fall into four classes of bilaterally symmetric pairs of neurons [the dorsal cephalic sensillum (CEP) (CEPD), ventral CEP (CEPV), anterior deirid sensillum (ADE), and posterior deirid (“postdeirid”) sensillum PDE] (Figure 1) (Sulston *et al.* 1975). All four neuron classes are ciliated mechanosensory neurons that form part of specific sensilla, the PDE, ADE, and CEPs (White *et al.* 1986). The four classes of dopaminergic neurons in *C. elegans* regulate a variety of behaviors, including mechanosensation, locomotion, habituation to mechanical stimuli, evaluation of food availability, swim to crawl transition, and spatial pattern selectivity (Chase *et al.* 2004; Han *et al.* 2017; Hills *et al.* 2004; Kindt *et al.* 2007; Sanyal *et al.* 2004; Sawin *et al.* 2000; Vidal-Gadea *et al.* 2011). Genetic screens for mutants that fail to produce terminally differentiated dopaminergic neurons have revealed that all four pairs of neurons are instructed by the same set of terminal selector-type transcription factors to adopt terminal dopaminergic neuron identity: the ETS domain transcription factor *ast-1*, the Distalless ortholog *ceh-43*, and one of several *C. elegans* Pbx genes (Doitsidou *et al.* 2008, 2013; Flames and Hobert 2009; Siehr *et al.* 2011). In the absence of these factors, dopaminergic neurons fail to initiate and maintain the terminal, dopaminergic differentiation program.

**Figure 1:**
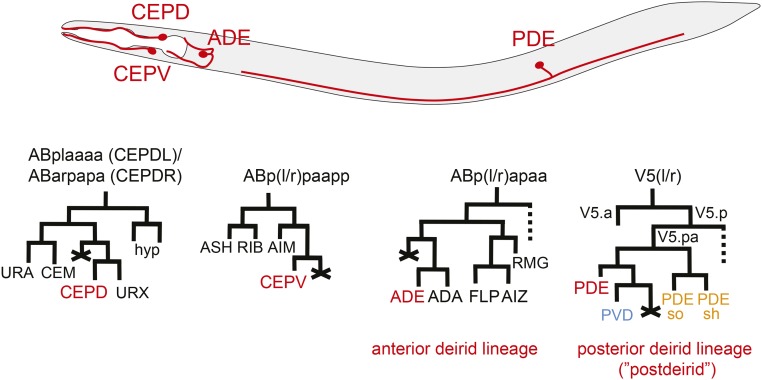
Dopaminergic neurons of the *C. elegans* hermaphrodite and their lineages. Lineage data comes from (Sulston and Horvitz 1977; Sulston *et al.* 1983). Dopaminergic neurons were first identified by Sulston *et al.* (1975). ADE, anterior deirid sensillum; CEP, cephalic sensillum; CEPD, dorsal CEP; CEPV, ventral CEP; PDE, postdeirid sensillum.

In spite of the striking similarities among dopaminergic neurons, the four different classes are born at different times during development, are situated in different parts of the nervous system, and derive from distinct neuronal lineages, as illustrated in Figure 1 (White *et al.* 1986). It is therefore to be expected that distinct factors operate in these distinct lineages to eventually specify terminal dopaminergic neuron identity. Indeed, classic screens for lineage mutants have uncovered the *C. elegans* homolog of Atonal, *lin-32*, as an essential regulator of the postdeirid lineage (Zhao and Emmons 1995). Intriguingly, *lin-32* has different effects on distinct dopaminergic neuron lineages. While the CEPDs also fail to be generated, the CEPVs, which arise from distinct neuroblasts (Figure 1), appear unaffected in *lin-32* loss-of-function mutants (Doitsidou *et al.* 2008).

Rather than screening for lineage patterns *per se*, we have initiated genetic screens for mutants in which dopaminergic neuron-specific identity markers fail to be properly expressed (Doitsidou *et al.* 2008; Nagarajan *et al.* 2014). Undertaking such screens, we have identified, as expected, *lin-32* mutant alleles (Doitsidou *et al.* 2008), terminal selectors for dopaminergic neuron identity (Doitsidou *et al.* 2013; Flames and Hobert 2009), and have shown that the *vab-3/Pax6* gene restricts the number of dopaminergic neurons produced (Doitsidou *et al.* 2008). In this paper, we describe a set of additional regulators of dopamine neuron lineage specification. We identify and characterize *ztf-6*, a C2H2 zinc finger transcription factor-encoding gene that acts in a subset of the dopaminergic neuron lineages to control the production of dopaminergic neurons.

## Materials and Methods

### Mutant isolation, mapping, and cloning

Transgenic worms carrying a chromosomally integrated *dat-1*::*gfp* reporter (*vtIs1*) were mutagenized using Ethyl Methanesulfonate (EMS) using standard protocols (Brenner 1974). Ensuing generations were screened for abnormal *dat-1*::*gfp* expression using a fluorescent dissecting microscope or the COPAS Biosort system (Doitsidou *et al.* 2008). *ot* alleles were isolated in the Hobert laboratory and *hu* alleles were isolated in the Korswagen laboratory (Soete 2007). All *hu* alleles were mapped and cloned by combined Hawaiian SNP mapping and whole-genome sequencing (WGS) or Variant Discovery Mapping (VDM), as previously described (Doitsidou *et al.* 2010; Minevich *et al.* 2012). The *ot280* allele was mapped prior to the appearance of the combined SNP/WGS modern methods (Doitsidou *et al.* 2010), using conventional high-throughput SNP mapping (Davis *et al.* 2005). After SNP mapping to the right arm of chromosome I (+5.06–+9.23 CM), *ot280* was whole-genome sequenced using an Illumina platform, followed by data analysis initially using MAQGene (Bigelow *et al.* 2009) and then reanalysis with CloudMap (Minevich *et al.* 2012). Data were filtered as previously described (Sarin *et al.* 2010). As a general rule, all mutant alleles were backcrossed a minimum of three times.

### Transgenic reporter strains

The *ztf-6*::*gfp* reporter strain *otEx6298* [*ztf-6*::*gfp*; *ttx-3*::*rfp*] was generated by *in vivo* recombination (Boulin *et al.* 2006). First, a genomic region from the first intron to the last exon of the *ztf-6* locus was amplified and fused to *gfp* using standard PCR fusion technology (Hobert 2002). The resulting fusion protein was co-injected with an amplicon that spanned a 4.7 kb sequence upstream of the first exon, the first exon, and the first intron. This amplicon overlapped by 50 bp with the PCR-fused amplicon to allow for *in vivo* recombination in the injected animals (Boulin *et al.* 2006). The following cellular identity markers were used to characterize the *ztf-6* mutant phenotype: *vtIs1* [*dat-1*::*gfp*; *rol-6*] (Nass *et al.* 2005), *vsIs33* [*dop-3*::*rfp*] (Chase *et al.* 2004), *otIs199* [*cat-2*::*gfp*; *rgef-1*::*dsRed2*] (Flames and Hobert 2009), *otIs355* [*rab-3*::*NLS*::*TagRFP*] (Doitsidou *et al.* 2013), *otIs14* [*zig-3*::*gfp*; *rol-6*] (Aurelio *et al.* 2002), *oyIs14* [*sra-6*::*gfp*; *lin-15*] (Troemel *et al.* 1995), *vsEx518*[*kcc-3*::*gfp*; *lin-15*(*+*)] (Tanis *et al.* 2009), *jcIs1* [*ajm-1*::*gfp*] (Mohler *et al.* 1998), *stIs10166* [*dpy-7p*::*HIS-24*::*mCherry + unc-119*(*+*)] (Murray *et al.* 2012), *qIs74*[*POP-1*::*GFP*] (Siegfried and Kimble 2002), and *stIs10226* [*his-72p*::*HIS-24*::*mCherry*::*let-858 3′ UTR + unc-119*(*+*)] (Murray *et al.* 2012).

### Lineage analysis

To create strains for suitable for time-lapse microscopy, mutant *ztf-6* animals carrying *vtIs1* were crossed with *stIs10166* [*dpy-7p*::*HIS-24*::*mCherry + unc-119*(*+*)] (Murray *et al.* 2012), a nuclear histone marker driven by the *dpy-7* promoter. The transgene expresses brightly in the hypodermal cells, but also moderately in the seam cells and postdeirid lineage in the L2 stage. The complete wild-type genotype was *stIs10166*; *sIs11337* [*rCesY37A1B.5*::*GFP + pCeh361*]; *ncIs13* [*ajm-1*::*GFP*] (Liu *et al.* 2005; McKay *et al.* 2003). We performed time-lapse imaging and microchamber fabrication as previously described (Gritti *et al.* 2016). Briefly, we used a Nikon Ti-E inverted microscope with a 60× magnification objective (Nikon Plan Apo 60×, NA = 1.4, oil immersion), with microchambers 195 × 195 μm in size. Transmission imaging was performed using a red LED (CoolLED pE-100 615 nm), while GFP fluorescence images were acquired using a 488 nm laser (Coherent OBIS LS 488-100) and mCherry fluorescence images were acquired using a 561 nm laser (Coherent OBIS LS 561). Images were acquired in a temperature-controlled room at 19° with sample temperature of 23°, and imaged every 20 min. Exposure time for experiments was 10 ms and ∼30 images were taken with a z-distance of 1 μm. Early divisions in the lineage (up to and including V5.pa and V5.pp) could be determined on both sides of the worm, but later divisions could only be visualized on the side closest to the objective. The Fiji distribution of ImageJ (Schindelin *et al.* 2012, 2015) was used for analysis and animals were straightened with the default ImageJ straighten macro for creation of the figures. The developmental stage of the animals was determined by observation of ecdysis in the transmitted light images. In *ztf-6* mutants showing incomplete postdeirid lineages, we ensured that *dat-1*::*gfp* expression was absent rather than delayed by verifying images in the late L2/start of L3.

### POP-1 localization

Mutant or control worms carrying the transgenes *qIs74*[*POP-1*::*GFP*] (Siegfried and Kimble 2002) and *stIs10226* [*his-72p*::*HIS-24*::*mCherry*::*let-858 3′ UTR + unc-119*(*+*)] (Murray *et al.* 2012) were bleached, and their eggs were collected and allowed to develop on OP50 plates at 20° for ∼22–26 hr. Worms were anesthetized with 10 mM levamisole and images were captured on a confocal microscope with a 100× magnification objective. ImageJ was used to quantify the average level of POP-1::GFP signal outlined by the localization of the nuclear marker *HIS-24:H2B*::*mCherry* at the most central z-stack containing the nucleus of the V5.pa and V5.pp daughter cells. The fluorescence in the anterior cell was divided by the fluorescence of the posterior cell of the pair to calculate the ratio of POP-1::GFP localization. Sister-cell pairs with fluorescence levels of 10% or less were considered equal/symmetric.

### Data availability

All data are represented in the paper's tables, figures and supplementary information. The timelapse imaging used for the lineage analysis presented here and the whole genome sequencing data that led to the identification of the phenotype causing variants can become available upon request.

## Results and Discussion

### Mutants with loss of the PDE postdeirid dopaminergic neurons

All dopaminergic neurons can be labeled in transgenic worms with a reporter construct that monitors expression of the dopamine reuptake transporter *dat-1* (Nass *et al.* 2002). Using this reporter transgene, we screened for EMS-induced mutants in which *dat-1*::*gfp* expression is lost. Our screens identified > 20 mutant strains that displayed a loss of *dat-1*::*gfp* expression in the postdeirid lineage. We identified the molecular lesions in nine of these strains through a combination of SNP mapping and WGS (Table 1).

**Table 1:** Proneural bHLH and mediator components affect neuron generation in the postdeirid

	Gene	Allele	Animals Expressing *dat-1*::*gfp* in PDE (%)*^a^*	Molecular Nature
Wild-type			100	
Proneural bHLH	*lin-32*	*ot263**^b^*	31	Noncoding point mutation, 60 bp upstream of the 5′ UTR. (LGX: 2,230,154 C > T), Genomic context: agacgaagctCcgcccacccg
		*hu72*	48	Missense: A91V in bHLH domain
		*hu75*	23	Missense: A91V in bHLH domain
	*hlh-2*	*hu82*	0	989 bp deletion (LGI: 7,201,151–7,202,139), 6,953 upstream of the 5′ UTR, cagaaaacatΔgaacgagaaa
Mediator	*cic-1*	*hu80*	0	Premature stop exon 2: W44Stop (LGIII: 2,401,021 C > T), acaattcgccCagaaaatatt
		*hu135*	25	Premature stop exon 6: W240Stop (LGIII: 2,395,807 C > T), cttcgacaagCcaagcttcca
		*tm3740**^c^*	59	Deletion allele
	*dpy-22*	*hu97*	16	Premature stop exon 9: Q1203Stop (LGX: 9,817,929 G > A), tgataggtttGtgctcggaat
		*os38**^c^*	21	Deletion allele*^d^*
	*cdk-8*	*hu96*	25	Premature stop exon 6: W227Stop (LGI: 8,703,554 G > A), attgacgtatGggctatcgga
		*tm1238**^c^*	52	Deletion allele

Coordinates from reference genome WS220. Genetic context is provided -the mutation is depicted in capital letter. PDE, postdeirid sensillum; bHLH, basic helix-loop-helix; LG, linkage group.

a Scored with *vtIs1* (*n* > 40).

b Previously called *dopy-6* (Doitsidou *et al.* 2008); phenotypic data were previously reported, numbers shown for comparison only.

c Another known allele of respective locus, scored for comparison to our alleles.

d (Yoda *et al.* 2005)

One set of mutants affect three subunits of the Mediator complex: *cdk-8* (cyclin-dependent kinase), *dpy-22* (also called MDT12), and *cic-1* (Cyclin C) (Table 1). Other available alleles of these Mediator complex components show the same phenotype (Table 1). *cdk-8*, *dpy-22*, and *cic-1* are all components of the kinase module of the Mediator complex (Grants *et al.* 2015). Generally, the Mediator complex is known to link a large number of distinct transcriptional regulatory complexes to RNA Polymerase II and has been implicated in a number of biological processes in multiple organisms, including in *C. elegans* (Grants *et al.* 2015). The mediator complex, including its kinase module, is broadly if not ubiquitously expressed (Steimel *et al.* 2013; Yoda *et al.* 2005) and, as expected, affects other lineages besides the PDE (including the Q lineage and the rays) (Soete 2007). We chose to not pursue its function further in the context of the postdeirid lineage.

Another set of mutants with loss of *dat-1*::*gfp* expression in the postdeirid affect two known proneural genes: *lin-32/*Atonal and *hlh-2*/Daughterless. As mentioned above, *lin-32* mutants were previously found to affect the generation of the postdeirid and we identified three new alleles of *lin-32*: *hu72* and *hu75* (missense mutations in the bHLH domain), and *ot263* (a presumptive *cis*-regulatory mutation) (Table 1). The *hu82* allele defined an unusual allele of the *hlh-2/*Daughterless locus. *hu82* fails to complement the hypomorphic *hlh-2*(*bx108*) allele (*bx108* homozygous animals display a wild-type postdeirid lineage, but only 11 out of 29 *bx108/hu82* heterozygote animals displayed a wild-type phenotype) and *hu82* is rescued by a fosmid that contains the *hlh-2* locus (five transgenic lines scored, all showing rescue). The involvement of *hlh-2* in postdeirid specification is expected, given that *hlh-2* is a well-established dimerization partner of *lin-32* (Portman and Emmons 2000). However, the recovery of an *hlh-2* allele from our screen was not anticipated because complete removal of *hlh-2* results in early lethality (Krause *et al.* 1990; Nakano *et al.* 2010), while *hu82* animals are fully viable even though they display a fully penetrant loss of *dat-1*::*gfp* expression. The likely reason for the lack of pleiotropies of *hu82* is that *hu82* does not affect the *hlh-2* coding region, but rather contains a 989 bp deletion 7 kb upstream of the *hlh-2* locus (Table 1), suggesting that *hu82* is a regulatory allele of *hlh-2* that affects its expression only in subsets of the cells that normally express *hlh-2*.

### ztf-6 mutants display altered patterns of dat-1 expression in a lineage-specific manner

**Figure 2:**
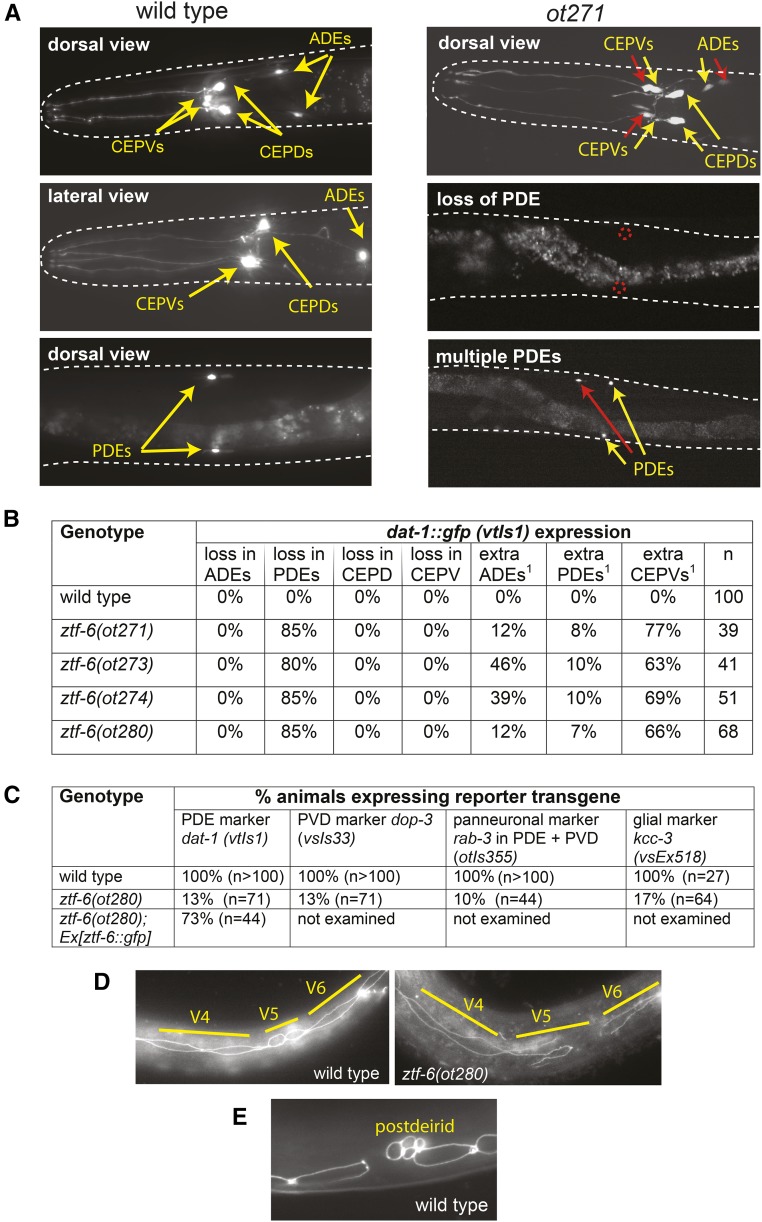
*ztf-6*(*ot271*) mutant animals show defects in lineages that produce dopaminergic neurons. (A) Expression of the dopamine transporter *dat-1*::*gfp* (*vtIs1*) in wild-type and *ztf-6* mutant backgrounds. Yellow arrows indicate dopaminergic cell bodies normally present, red arrows indicate extra cells generated, and the dotted red circles expected positions of missing cells. (B) Quantification of defects. “^1^” indicates cells located in close proximity to the original neuron. (C) Terminal markers fail to be expressed in the postdeirid lineage of *ztf-6* mutants. (D) Seam cell defects in *ztf-6* mutants. Seam cells are shown in early larval stages when V cells divide. (E) Characteristic cluster of neuro- and glioblast cells of the postdeirid lineage, observed in wild-type animals. These clusters are difficult to observe in *ztf-6* mutants. ADE, anterior deirid sensillum (ADE); CEP, cephalic sensillum; CEPD, dorsal CEP; CEPV, ventral CEP; PDE, postdeirid sensillum.

We focused our attention on four mutant strains that define a previously nondescribed complementation group. Initially, we called the locus defined by this complementation group *dopy-1* but, because of the molecular identity of the mutants (described below), we renamed the locus *ztf-6*. Expression of *dat-1*::*gfp* in the PDE neurons, which form part of the postdeirid structure, is lost in most of the *ztf-6* mutant animals (Figure 2, A and B). In a subset of the infrequent cases where *dat-1*::*gfp* expression in the PDE neurons is not turned off, we also observe ectopic *dat-1*::*gfp* expression in the postdeirid lineage (Figure 2B).

Loss of *dat-1*::*gfp* expression in the PDE neurons of the postdeirid lineage of all *ztf-6* mutant alleles is paralleled by a loss of another dopaminergic marker (tyrosine hydroxylase *cat-2*::*gfp*; data not shown). Loss of PDE identity is likely a reflection of gross misspecification or entire loss of the postdeirid lineage, since the expression marker for all other cells in the postdeirid lineage are lost in *ztf-6* mutants (Figure 2C). These affected markers include a reporter for the glutamatergic PVD neurons (*dop-3*) (Chase *et al.* 2004), a panneuronal marker (*rab-3*), and a marker for the glial cells generated by the lineage (*kcc-3*) (Bellemer *et al.* 2011) (Figure 2C).

*ztf-6* is not only required to generate the postdeirid lineage from the V5 ectoblast, but is also required for the specification of the PDE neurons in the ectopic postdeirid lineage observed in *lin-22/hairy* mutants (Wrischnik and Kenyon 1997), as assessed by the analysis of *lin-22*; *ztf-6* double mutants (Table 2).

Using *ajm-1*::*gfp* to mark the outline of dividing V5 blast cells, we examined the fate of the V5 cells that generate the postdeirid (Figure 1) in more detail. The shape and mutual attachment of dividing V5 cells appear abnormal in most *ztf-6* animals scored (Figure 2D). Moreover, the characteristic cluster of cells that generate neurons and glia cells, observed in a specific time window in wild-type animals (Figure 2E), is difficult to observe in *ztf-6* mutants, suggesting that the postdeirid lineage is not formed.

**Table 2:** Genetic interaction of *lin-22* and *ztf-6*

Genotype	% Lineages with *vtIs1*[*dat-1*::*gfp*] Expression (% Lineages Producing Multiple PDEs)						
	V1	V2	V3	V4	V5	V6	*n* (Worms)
Wild-type	0	0	0	0	100	0	120
*lin-22*(*n372*)	17 (0)	78 (1)	84 (1)	87 (1)	100 (0)	0	40
*ztf-6*(*ot271*)	0	0	0	0	15 (8)*^a^*	0	39
*lin-22*(*n372*); *ztf-6*(*ot271*)	27 (8)	45 (29)	56 (35)	42 (24)	22 (8)	0	40

**Figure 3:**
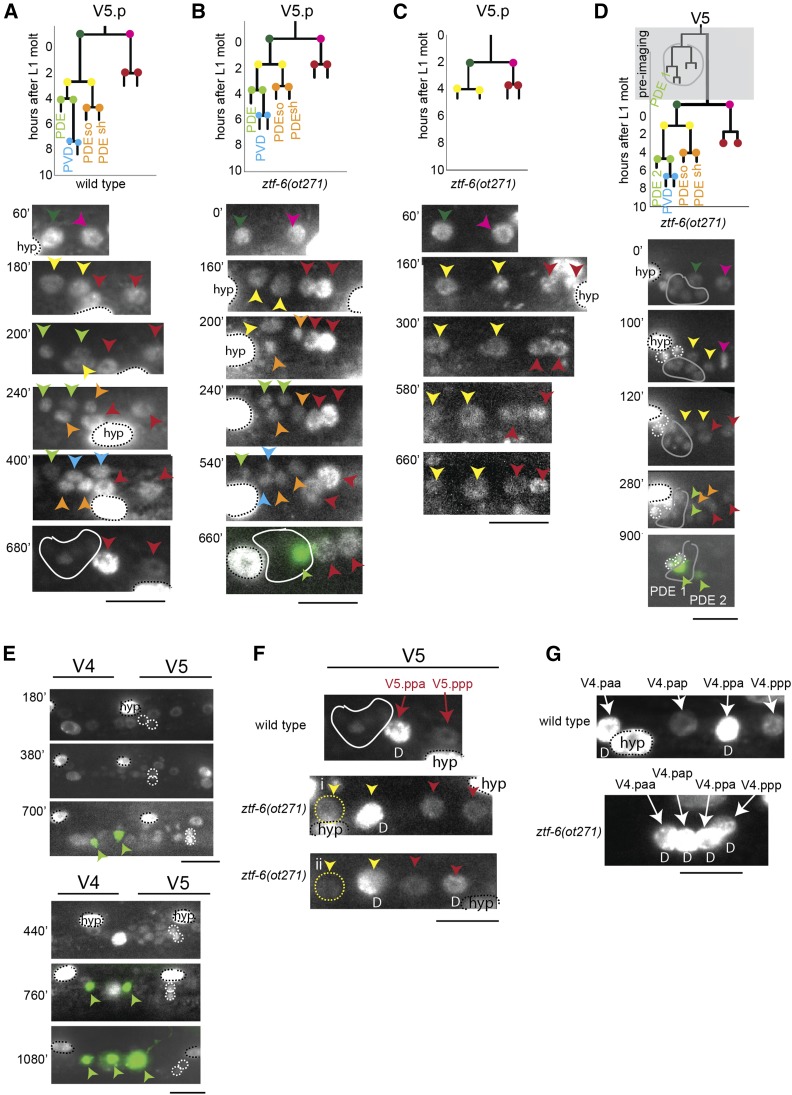
Lineage analysis of the postdeirid of *ztf-6* mutants. Black bar, 10 μm in all panels. Presence of large hypodermal nuclei not related to the postdeirid or L2 seam cell lineage are designated as “hyp” in the initial image and circled with a black dashed line. The unilateral SDQL and PVM neurons are circled in a dashed white circle, when present, to differentiate them from cells in the postdeirid lineage. (A–C) Examples of V5.p lineages in wild-type and *ztf-6* animals. Nuclear marker expression (*stIs10166*) in the V5.p lineage was followed over time to determine cell division times and postdeirid phenotypic information. Cell colors in the lineage chart correspond to colored arrows and cells in the images. Postdeirid lineages in older animals with faded nuclear marker expression are circled in a solid white line, due to the loss of expression of the hypodermal fate marker later in development. If present in the genetic background, *vtIs1* (*dat-1*::*gfp*) is shown overlaid in green. (D) Temporal defect in postdeirid lineage formation in a *ztf-6*(*ot271*) mutant. An ectopic postdeirid lineage contained by a gray outline was already present at the start of the L2 stage before data acquisition was initiated. During the L2 stage, V5.p produced a postdeirid lineage, resulting in two *dat-1*::*gfp* expressing neurons. *dat-1*::*gfp* expression turned on at similar times for both postdeirid sensillum (PDE) neurons in the late L2 stage. (E) Spatial patterning defects in two different *ztf-6*(*ot271*) animals showing spurious cell divisions in the V4.p lineages. Ectopic *dat-1*::*gfp* expressing cells appear in the V4 region of both animals due to increased cell division. (F) Late L2 stage wild-type and *ztf-6*(*ot271*) animals showing reversed asymmetry or lack of asymmetry of *dpy-7* expression in V5.p mutant lineages. Near the end of the L2 stage (10–14 hr after start of L2 stage), the remaining cells in the V5.p lineage were scored for strong *dpy-7* expression as a proxy for hypodermal fate, with the D notation representing the presumptive differentiated (hypodermal) cell fate based on relative expression level between seam cells and existing hypodermal nuclei. Wild-type image directly from (A) 680’ time point, showing the proper *dpy-7*/differentiation pattern of the V5.ppa and V5.ppp cells. Mutant animal (i) shows lack of asymmetry, with neither seam cell taking the hypodermal fate, while mutant animal (ii) shows reversed asymmetry. Some seam cells are circled with a dashed yellow line for easier visualization. (G) Late L2 stage wild-type and *ztf-6*(*ot271*) mutant V4.p lineages showing *dpy-7* expression patterns. In the wild-type animal, expression levels in the V4.paa and V4.ppa cells indicate the hypodermal fate, marked with a D (differentiated). In the mutant, all of the V4 seam cells acquired the hypodermal fate instead of only the anterior of each pair. Also, note the abnormally clumped spacing of the nuclei in the mutant.

PDE, postdeirid sensillum.

a Some of the ectopically generated PDEs may arise from the V4 lineage (see Figure 3E).

### Postdeirid lineage analysis of ztf-6 mutants

The loss of the postdeirid lineage prompted us to investigate the *ztf-6* mutant phenotype in more detail. Due to the length of time that the postdeirid lineage takes to develop, and the number of lineages we wished to follow, we turned to a novel time-lapse microscopy technique that allowed us to carefully track development in freely moving and normally developing animals (Gritti *et al.* 2016). We performed lineage analysis focusing on the V5.p cell lineage, starting in the late L1/early L2 stage when the cell divides to form V5.pa and V5.pp. We used a *dpy-7* promoter-driven nuclear histone marker that is moderately expressed in seam cells and the postdeirid lineage, and highly expressed in hypodermal nuclei, thus providing information on both lineage and the assignment of hypodermal cell fate. With our marker, it was possible to observe all cell divisions that constitute the postdeirid lineage in wild-type worms (Figure 3A). In the *ztf-6*(*ot271*) mutant, we found that the postdeirid lineage divisions occurred normally in 5/26 of the V5.p lineages (Figure 3B). However, in the majority of the V5.p lineages,(16/26), cell divisions were delayed and eventually became arrested after the division of the V5.pa cell (Figure 3C). Additionally, the mutation acted independently in the left and right lineages in the animal, since comparing the formation of the postdeirid lineages on both sides of a single animal showed that cell divisions in the mutant lineage were delayed up to 2 hr compared to the normally forming postdeirid lineage that developed on the other side (Supplemental Material, Figure S1). Due to anatomical abnormalities or defects that occurred earlier in development, 2/26 lineages could not be scored.

Along with the loss of the postdeirid lineage due to arrested cell divisions, we also observed a minority of cases (3/26) in which the *ztf-6*(*ot271*) mutation exhibited a gain-of-function phenotype in which extra *dat-1*::*gfp* expressing neurons were generated. In particular, in one animal, we observed that an entire postdeirid lineage had already been generated during the L1 stage of development, before we had started our time-lapse data collection, resulting in an animal with two postdeirid lineages and two *dat-1*::*gfp*-expressing cells by the end of the L2 stage (Figure 3D). In addition to this temporal defect, we also detected expansions of the postdeirid lineage pattern occurring during the L2 stage that resulted in multiple *dat-1*::*gfp*-expressing neurons and an excess of other cells generated during the cell divisions, which likely originated from the V4 lineage (Figure 3E). Due to the number of cells in a small area and their divisions occurring rapidly within a short period of time, the exact lineage details in these animals could not be followed in detail, but the excess of generated cells in V4 and V5 lineages demonstrates the tendency for spurious cell divisions to occur in the *ztf-6* mutant.

Additionally, we often observed improper expression of the *dpy-7* hypodermal cell fate marker in the mutant, suggesting that the cells had improperly differentiated, although the pattern and extent of this phenotype was highly stochastic between animals. This was true of the V5.ppa and V5.ppp cells as well, which are not directly involved in the development of the postdeirid lineage (Figure 3F). Lineages other than V5.p also exhibited variable abnormalities in their expression patterns of *dpy-7* after the divisions of Vn.pa and Vn.pp (Figure 3G), suggesting that *ztf-6* is important for seam cell identity and proper cell fate patterns in seam cell lineages outside of V5.

### Loss of canonical asymmetric cell division in ztf-6 mutants

**Figure 4:**
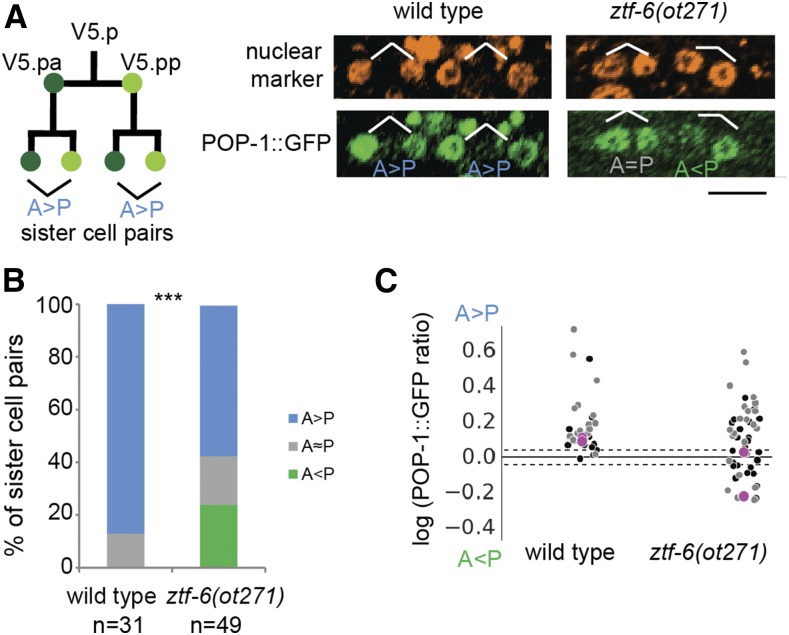
*ztf-6* affects asymmetric POP-1 localization. (A) Schematic of the V5.p asymmetric divisions before specification of the postdeirid lineage. The anterior and posterior sister cell pairs resulting from the division of V5.pa and V5.pp were analyzed for asymmetric POP-1 localization in the mid-L2 stage. POP-1::GFP levels were quantified and the asymmetric distribution was determined in wild-type and *ztf-6* mutant animals. Black bar, 12 μm. (B) Comparison of the distribution of POP-1::GFP in V5.pa and V5.pp daughter cells. There is an increase in sister cells pairs with inverted POP-1 localization (A < P) in the mutant compared to control (*P* < 0.003, Fisher Exact Test). (C) Comparison of the logarithm of the ratio of POP-1::GFP between sister cells. Each marker represents one sister cell pair, with black representing anterior pairs and gray representing posterior pairs (data plotted from (B)). Magenta dots in the plot correspond to the sister cell pairs shown in (A). Dashed black lines indicate the ratios between which we considered the sister cells pairs symmetrical. GFP, green fluorescent protein.

The variability of the phenotypes observed in the *ztf-6* mutant along with the abnormalities in cells expressing the hypodermal fate marker were reminiscent of Wnt mutations on seam cell polarity and fate (Yamamoto *et al.* 2011), which prompted us to characterize the Wnt/β-catenin asymmetry pathway in *ztf-6* mutants. The Wnt/β-catenin asymmetry pathway is responsible for proper seam cell fate and other asymmetric divisions by regulating the distribution of SYS-1/β-catenin and POP-1/TCF in a reciprocal manner (Nakamura *et al.* 2005; Takeshita and Sawa 2005). After an initial seam cell division at the beginning of the L2 stage, Vn.pa and Vn.pp undergo an asymmetric cell division, in which POP-1 is preferentially localized to the anterior sister cell, which we confirmed in wild-type animals (Figure 4A) (Lin *et al.* 1998). However, POP-1 asymmetric localization in the *ztf-6*(*ot271*) mutant background was less robust than in wild-type animals, with > 20% of sister cells showing an inverted pattern of POP-1 asymmetry, with the posterior sister cell having higher POP-1 levels than the anterior sister cell, something never observed in wild-type animals (Figure 4, B and C). Similar findings were observed in the V4 and V6 lineages (data not shown). Together, these results suggest that ZTF-6 helps to maintain the robustness of the Wnt/β-catenin asymmetry pathway.

### ztf-6 mutants show distinct phenotypes in distinct dopaminergic lineages

In contrast to the loss of *dat-1*::*gfp* expression in the PDE neurons, *dat-1*::*gfp* expression in CEPD neurons is unaffected in all *ztf-6* mutant alleles (Figure 2, A and B). The ADE also shows normal *dat-1:gfp* expression in *ztf-6* mutants. Interestingly, however, *dat-1* expression is also observed in one to two neurons that are localized in very close apposition to the ADE (Figure 2, A and B). Similarly, neurons with ectopic *dat-1*::*gfp* expression are observed in close proximity to the CEPV neurons (Figure 2, A and B).

To test whether the ectopic CEPV neurons are generated as a result of the survival of the normally dying sister of CEPV neurons (see lineage in Figure 1), we examined whether the prevention of cell death, observed in *ced-4* mutant animals, leads to the undead cell indeed adopting a dopaminergic identity, as assessed by *dat-1*::*gfp* expression. As deduced by the observation of additional *dat-1*::*gfp*-positive cells in close apposition to CEPV on both sides of the animals (CEPVL and CEPDR; see lineage in Figure 1), we indeed find this to be the case in *ced-4* mutants. We then generated a *ztf-6*; *ced-4* double mutant strain and found an additive phenotype in these double mutant animals, suggesting that the ectopic CEPV neurons are not the result of the CEPV sister cells surviving. The quantification is as follows: *ztf-6*(*ot280*), on average 1.08 ectopic *dat-1*::*gfp*(*+*) cells; *ced-4*(*n1162*), 1.95 ectopic *dat-1*::*gfp*(*+*) cells; and *ztf-6*(*ot280*), *ced-4*(*n1162*), on average 2.69 *dat-1*::*gfp*(*+*) cells (n ≥ 40 for each genotype) (Figure S2).

We also find that the fate of other neurons in the CEPV lineages, specifically the AIM and ASH cells generated by the same lineage (see lineage in Figure 1) are specified normally in *ztf-6* mutants (as assessed with an AIM-expressed *zig-3*::*gfp* reporter and an ASH-expressed *sra-6*::*gfp*; data not shown). Given the proximity of the ectopic *dat-1*::*gfp*(*+*) cell to the normal CEPV, we therefore consider it most likely that in *ot280* mutants one round of extra cell division of the CEPV neurons occurs. We were not able to directly observe such additional division with lineaging techniques due to the vigorous movement of embryos in late embryonic stages.

### ztf-6 encodes a C2H2 zinc finger transcription factor

**Figure 5:**
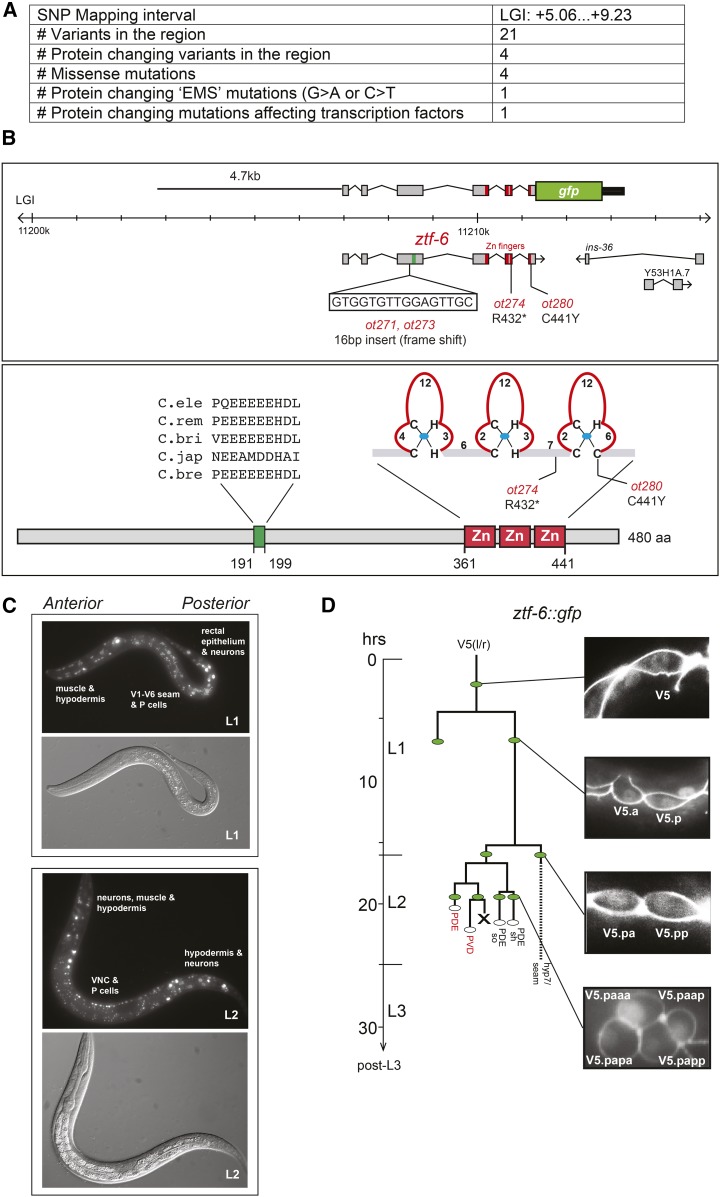
Cloning and expression of *ztf-6*. (A) Summary of whole-genome sequence data of *ztf-6*(*ot280*). (B) *ztf-6* gene locus, reporter gene used in this study, and ZTF-6 protein structure. Nucleotide changes are as follows: *ot271* and *ot273*, 5′-TGGCAGGAAGTGGTGTTGGAGTTGCCGAACGGCT-3′ (underlined: 16 bp insertion); *ot274*, C to T in 5′-ATGGTCTTCGATTGCATAGA-3′; and *ot280*, G to A in 5′-AGAAATCCAGCATGCATGGAAG-3′. (C) Expression of *ztf-6*::*gfp* (*otEx6298*) at the L1 and L2 stages. Note that some autofluorescence is visible as small bright dots in the middle part of the body. (D) *ztf-6*::*gfp* expression in the V5 lineage. Animals were staged by egg-laying and analysis at different stages, as indicated. EMS, Ethyl Methanesulfonate; SNP, single nucleotide polymorphism; VNC, ventral nerve cord.

We mapped the *ot280* mutation to a small interval on linkage group I using conventional SNP mapping approaches and then sequenced the genome of *ot280* mutant animals using Illumina technology (see *Materials and Methods*). Among the sequence variants present in this genetic interval, four affect amino acids in protein coding genes and only one affects a transcription factor (Figure 5A), the previously uncharacterized *ztf-6* gene. We Sanger-sequenced the *ztf-6* locus in all four available allelic mutant strains and found mutations in the *ztf-6* locus in each allele (Figure 5B). The PDE phenotype of *ot280* mutants can be rescued by a piece of genomic DNA that only contains the *ztf-6* locus (Figure 2C).

The ZTF-6 protein contains three zinc finger domains at its C-terminus, two C2H2 fingers and one C2HC finger, and a conserved acidic domain possibly involved in transcriptional activation. The *ot271* and *ot273* alleles contain identical short insertions at the beginning of the locus that result in a frameshift and premature stop before the three zinc finger domains of *ztf-6*, suggesting that these alleles are null alleles (Figure 5B). The *ot274* mutant contains a nonsense substitution before the third zinc finger. The last, somewhat more unusual zinc finger (a C2HC finger), is essential for protein function since one allele, *ot280*, disrupts the last cysteine of this finger (Figure 5B).

While there are ∼200 predicted C2H2 proteins encoded in the *C. elegans* genome (Knight and Shimeld 2001), there are no clear ZTF-6 paralogs in the *C. elegans* genome. There are clear orthologs of *ztf-6* in other *Caenorhabditis* species that display sequence similarity throughout the entire proteins (and close to 100% sequence identity among the three zinc fingers). As in *C. elegans*, several other nematode species that contain *ztf-6* contain no *ztf-6* paralogs, with the exception of *C. brenneri*, which contains two *ztf-6* paralogs, each containing the same characteristic last C2HC zinc finger. No ZTF-6 relatives are present in the currently available genome sequences of other non-Rhabditis nematodes, such as *Brugia malayi* or *Pristionchus pacificus*, nor in arthropods or chordates.

### Expression pattern of ztf-6

We generated a reporter construct that contains the *ztf-6* locus with all exons and introns and 4.7 kb of sequences upstream of the start codon (Figure 5A). This construct (Figure 5B) is able to rescue the *ztf-6*(*ot280*) mutant phenotype (see data in Figure 2C). Expression is first observed in late embryogenesis (threefold stage) (data not shown). In early larval stages, the *ztf-6*::*gfp*-expressing cells include head hypodermal cells, head muscle cells, neurons, and ectodermal blast cells along the body (all P and all V cells) and in the tail (Figure 5C). Starting in the L2 stage, additional neurons in P cell-derived ventral cord motor neurons express *ztf-6*::*gfp* (Figure 5C).

Because of the postdeirid loss phenotype of *ztf-6* mutants, we examined the postdeirid lineage in more detail. We observe *ztf-6* expression in the V5 cell of freshly hatched L1 animals. Upon division of the V5 cell into a posterior and anterior daughter, we observe expression in both the anterior and posterior daughters of the V5 cell (Figure 5D). The descendent of the posterior V5.p daughter, V5.pa (the founder of the postdeirid lineage), and V5.pp also continue to express *ztf-6*::*gfp*. Within the V5.pa lineage, expression of *ztf-6*::*gfp* is retained in the blast cells that generate the glial cells and the PDE and PVD neurons (Figure 5D). No expression is detected at later stages in this lineage.

### Conclusions

Through genetic screens for mutants in which a dopaminergic cell fate marker is aberrantly expressed, we identified a novel, previously uncharacterized zinc finger transcription factor, *ztf-6*, as being required to produce the proper set of dopaminergic neurons in the *C. elegans* nervous system. *ztf-6* has different effects on the distinct lineages that produce dopaminergic neurons in the PDE, ADE, and CEPs. In one lineage, it is required for the generation of the entire lineage (PDE), likely by controlling the differential activity of a binary lineage patterning system (Wnt/POP-1). In another lineage, *ztf-6* appears to have no role (CEPD), and in two other lineages *ztf-6* restricts the number of dopaminergic neurons generated. There are some similarities in this phenotypic spectrum of *ztf-6* mutants with the phenotypic spectrum of animals that lack the bHLH factor *lin-32/*Atonal, particularly in the deirid lineages. Both *lin-32* and *ztf-6* are proneural genes in the postdeirid lineage. In the anterior deirid lineage we previously noted that, like in *ztf-6* mutants, ectopic *dat-1*::*gfp* neurons are produced in *lin-32* mutants (Doitsidou *et al.* 2008). However, *lin-32* null mutants do not phenocopy the ectopic *dat-1*::*gfp* phenotype of the CEPV lineage of *ztf-6* null mutants. And, while *ztf-6* null mutants show no defects in *dat-1*::*gfp* expression in CEPD, the CEPD neuron are not generated in *lin-32* mutants (Doitsidou *et al.* 2008). Taken together, *ztf-6* and *lin-32* function appears to be related on some, but clearly not all, dopaminergic neuron-producing lineages.

The loss of *ztf-6* showed conflicting phenotypes in the PDE: most lineages displayed a loss-of-function phenotype, but some lineages showed a gain-of-function phenotype resulting in multiple *dat-1*::*gfp*-expressing neurons. Additionally, these were often accompanied by reversed or asymmetric hypodermal cell fate choices in other cells outside of the postdeirid lineage. Occasional loss of normal Wnt/β-catenin asymmetry in the postdeirid precursors could cause cell fate transformations depending on the levels of POP-1 in the cells and whether the localization is symmetrical or reversed, such as in V5.pp/pa daughter cells. *ztf-6* likely has other roles that affect the generation of the postdeirid lineage outside of the Wnt/β-catenin pathway, since errors in the lineage were more frequent than an absence or reversal of POP-1 asymmetry. For example, delayed cell division times in the lineage, the formation of the postdeirid lineage at the inappropriate developmental stage, or presence of the postdeirid lineage from the V4 lineage seem unlikely to arise directly from POP-1 asymmetry. Multiple targets of ZTF-6 are not unexpected due to its identity as a transcription factor. In addition, we detected defects in the characteristic hypodermal patterning of nonpostdeirid-containing seam cell lineages, such as the V4 lineage. In combination with results showing ectopic *dat-1*::*gfp*-expressing neurons originating from the V4 region, and the ability for a *ztf-6* mutant allele to rescue ectopic postdeirid lineages in the *lin-22*(*n372*) mutant background, these observations suggest that *ztf-6* may regulate all or most seam cell lineages, but with a more complicated role in the proper patterning of V5. Overall, ZTF-6 acts to stabilize and ensure that the postdeirid lineage is generated at the proper time and position.

ZTF-6 is one of ∼200 C2H2 zinc finger transcription factors encoded in the *C. elegans* genome, but appears to exist only in the Rhabditidae family of nematodes. This observation is in line with the general notion that, unlike other transcription factor families (*e.g.*, the homeobox family), C2H2 zinc finger transcription factors have undergone extensive species-specific expansion across the animal kingdom (Knight and Shimeld 2001). Our identification of a neuronal role for an orphan, nonconserved C2H2 zinc finger transcription factor contributes to the functional deorphanization of the many nonconserved C2H2 zinc finger transcription factors in the *C. elegans* genome. Invertebrate-specific or even nematode-specific C2H2 zinc finger transcription factors have also been uncovered by other screens for neuronal cell fate decisions (Baum *et al.* 1999; Chang *et al.* 2003; Johnston *et al.* 2006; Johnston and Hobert 2005; Zhang *et al.* 2011), suggesting that species-specific C2H2 zinc finger expansion may relate to species-specific nervous system features.

## Supplementary Material

Supplemental material is available online at www.g3journal.org/lookup/suppl/doi:10.1534/g3.117.300132/-/DC1.
