# Supplementary material for: A Caenorhabditis elegans Zinc Finger Transcription Factor, ztf-6, Required for the Specification of a Dopamine Neuron-Producing Lineage
Source: G3 (Bethesda). 2018 Jan 4;8(1):17–26. doi: 10.1534/g3.117.300132 (PMC5765345; doi:10.1534/g3.117.300132)
Supplement: Supplementary file 2 [file 17FigureS2.pdf]

A.

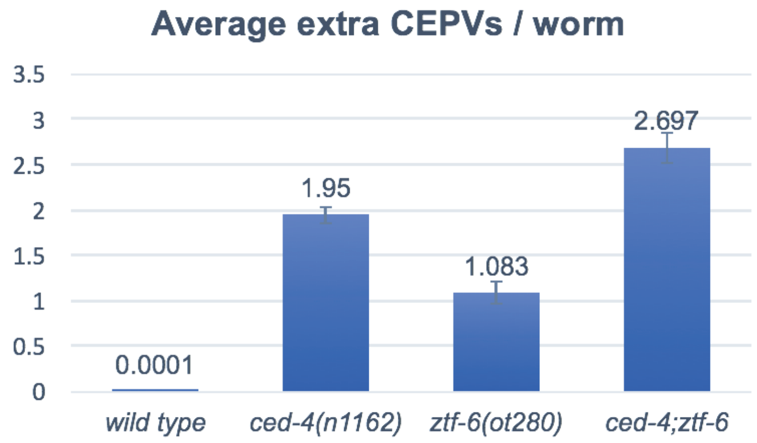

B. **Types of CEPV phenotypes in *ced-4;ztf-6* mutants**

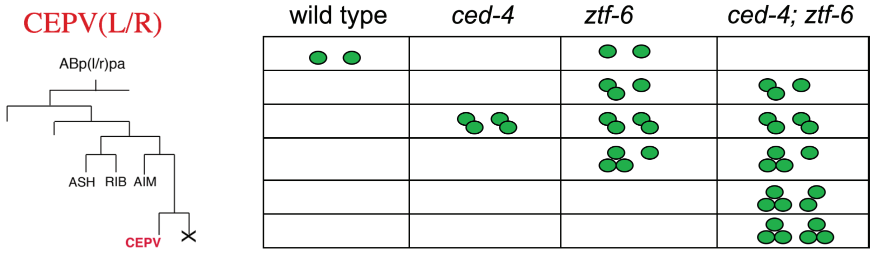

Supplementery Figure 2: Phenotypic analyis s of *ced-4;ztf-6* double mutants
A. Quantification of extra CEPVs (on average per worm) in *ced-4(n1162), ztf-6(ot280)* and double mutants. Error bars represent standard error of the mean. n=40-50
B. Left: lineage diagram of CEPV illustrating the dying sister cell. Right: Schematic representation of the various CEPV phenotypes met in *ced-4, ztf-6* and double mutants. The phenotype suggests that the extra CEPVs preseint in *ztf-6* mutants are not due to the failure of the sister cell to undergo apoptosis.
