# Supplementary material for: A Caenorhabditis elegans Zinc Finger Transcription Factor, ztf-6, Required for the Specification of a Dopamine Neuron-Producing Lineage
Source: G3 (Bethesda). 2018 Jan 4;8(1):17–26. doi: 10.1534/g3.117.300132 (PMC5765345; doi:10.1534/g3.117.300132)
Supplement: Supplementary file 1 [file 17FigureS1.pdf]

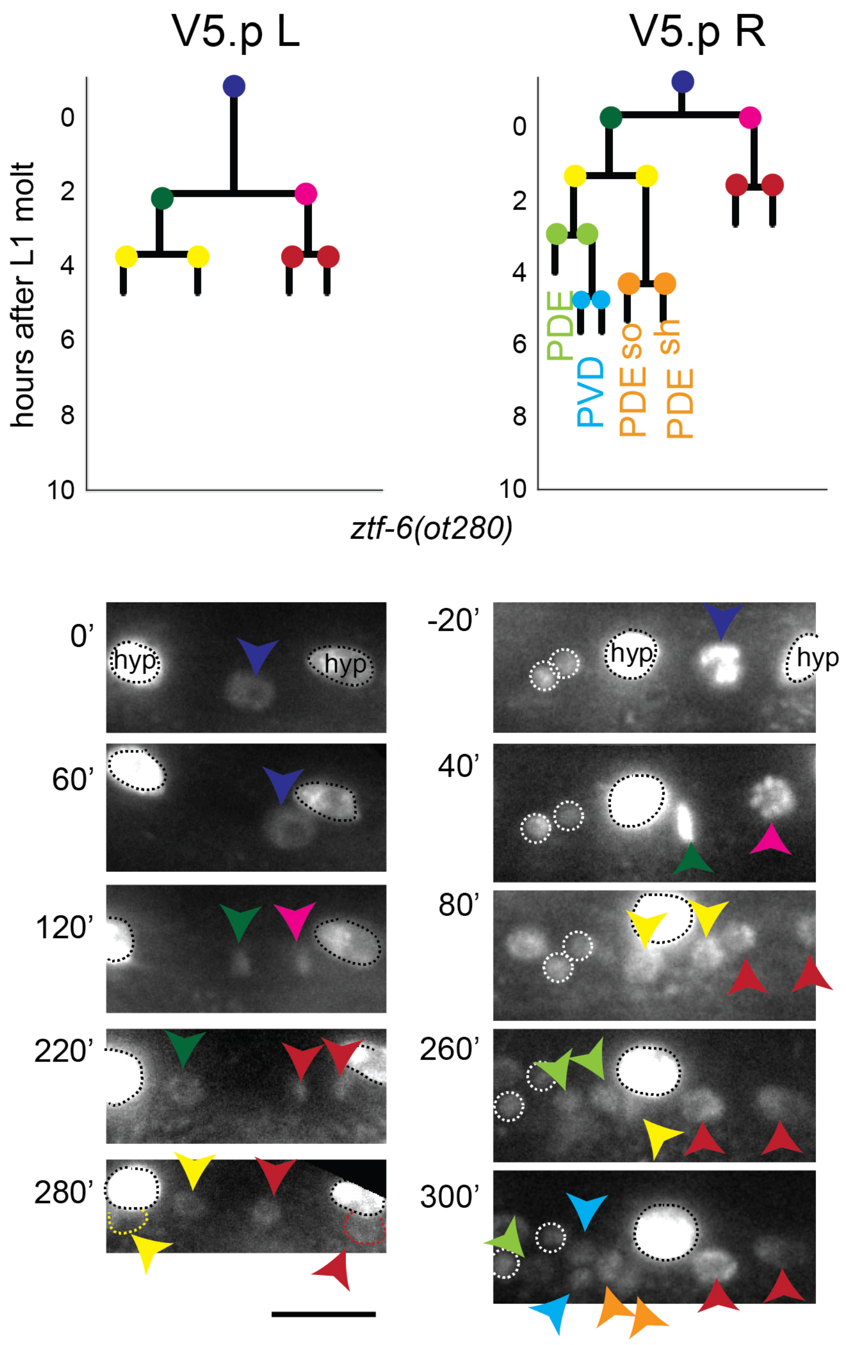

Supplementary Figure 1:
Independence of the mutant phenotype in the left and right postdeirid lineages

Example of the left and right postdeirid lineages in a *ztf-6(ot280)* animal. Nuclear marker expression (*stIs10166*) in the V5.p lineage was followed over time on each side to determine cell division times and postdeirid phenotypic information. Note the delay in the initial cell divisions between the left and right sides of the animal: the abnormal side had cell division delays of approximately two hours compared to the side that developed normally. Cell colors in the lineage chart correspond to colored arrows and cells in the images. The SDQL and PVM neurons (which appear on only one side of the animal and are not part of postdeirid lineage) are circled in a dashed white circle to differentiate them from cells in the postdeirid lineage. Presence of large hypodermal nuclei not related to the postdeirid or L2 seam cell lineage are designated as “hyp” in the initial image and circled by a black dashed line. Some seam cells are circled with a dashed yellow or red line for easier visualization. Black scale bar represents 10 μm.
